# Correlation Analysis of EV71 Detection and Case Severity in Hand, Foot, and Mouth Disease in the Hunan Province of China

**DOI:** 10.1371/journal.pone.0100003

**Published:** 2014-06-18

**Authors:** Li-Dong Gao, Shi-Xiong Hu, Hong Zhang, Kai-Wei Luo, Yun-Zhi Liu, Qiao-Hua Xu, Wei Huang, Zhi-Hong Deng, Shuai-Feng Zhou, Fu-Qiang Liu, Fan Zhang, Yu Chen

**Affiliations:** Hunan Provincial Center for Disease Control and Prevention, Hunan, China; University of Illinois at Chicago, United States of America

## Abstract

An increase in the incidence of hand, foot and mouth disease (HFMD) cases has been observed in the Hunan province of mainland China since 2009 with a particularly higher level of severe cases in 2010–2012. Intestinal viruses of the picornaviridae family are responsible for the human syndrome associated with HFMD with enterovirus 71 (EV71) and Coxsackievirus A16 (Cox A16) being the most common causative strains. HFMD cases associated with EV71 are generally more severe with an increased association of morbidity and mortality. In this study, the etiology surveillance data of HFMD cases in Hunan province from March 2010 to October 2012 were analyzed to determine if there is a statistically relevant linear correlation exists between the detection rate of EV71 in mild cases and the proportion of severe cases among all HFMD patients. As the cases progressed from mild to severe to fatal, the likelihood of EV71 detection increased (25.78%, 52.20% and 84.18%, respectively). For all cases in the timeframe evaluated in this study, the presence of virus was detected in 63.21% of cases; among cases showing positivity for virus, EV71 infection accounted for 50.14%. These results provide evidence to support the observed higher morbidity and mortality associated with this outbreak and emphasizes the importance of early detection in order to implement necessary prevention measures to mitigate disease progression.

## Introduction

Hand, foot and mouth disease (HFMD) is a moderately contagious viral illness that commonly affects infants and children; its initial symptomology is characterized by fever, reduced appetite and sore throat and mouth, which is followed by a skin rash with flat red spots that develops on the palms of the hands and soles of the feet. [1] Outbreaks of HFMD are the result of many factors, including multiple infection sources, complex and various transmission styles, susceptible populations, and a lack of an effective vaccine. [2] Currently, most reports regarding HFMD outbreaks have concentrated on the characteristics associated with a particular epidemic and potential individual risk factors for increased severity of disease. [3]–[5] The number of studies that has focused on mitigation of early disease to prevent the development of severe and/or fatal cases is limited.

Enterovirus 71 (EV71) and Coxsackievirus A16 (Cox A16) are considered to be the predominant pathogens responsible for the reported epidemics of HFMD. [6], [7] EV71 belongs to the genus *Enterovirus* in the *Picornaviridae* family and was first successfully isolated in 1969; since then, this virus has been found to be associated with higher virulence and potential for neurological disease. [8] [9] Previous studies have directly linked EV71 to the development of severe cases of HFMD, which often result in death. [8] [9] In this study, the etiological surveillance data of HFMD, gathered in the Hunan province from March 2010 through October 2012, were analyzed to determine if a correlation exists between the detection rate of EV71 in mild cases and the proportion of severe cases among all HFMD patients.

## Materials and Methods

### Diagnostic criteria

Cases of HFMD were classified as mild or severe according to the diagnostic criteria established in the “Hand, Foot and Mouth Disease Control and Prevention Guide” (2009) and in the “Hand, Foot and Mouth Disease Treatment Guidelines” (2010) published by the Ministry of Health of China. [10],[11] Briefly, the diagnosis standard of a mild case of HFMD includes fever with the presence of a skin rash on hands, feet, mouth, or buttocks. A severe case of HFMD is defined by presentation of additional neurological, cardiogenic or pulmonary disease. All cases were also tested by laboratory methods to determine the characteristic abnormal findings for hyperleukocytosis and hyperglycemia, cerebrospinal fluid parameters, and on imaging analyses, including electroencephalogram, cerebral spinal magnetic resonance, chest X-ray and ultrasonic cardiogram. Definitive diagnosis was made upon pathogen detection. At least five mild cases were detected in each district per month upon an initial visit to a public health facility; all severe and fatal cases were selected for inclusion in the detection analysis in this study.

The study was approved by institutional review board (IRB) of Hunan Centers for Disease Control and Prevention (Hunan CDC), and was conducted in compliance with the Helsinki Declaration. Written informed consent was obtained from all study participants. All patient data were anonymized.

### Pathogen detection

Case specimens (feces, throat swabs, rectal swabs, ulcer fluid, and cerebrospinal fluid) were collected from March 2010 through October 2013 and provided to us by provincial inspection agencies from the 14 cities in Hunan, by the Center for Disease Control (CDC) locations in Hunan province as well as the Hunan Provincial Children’s Hospital. We forwarded the specimens to the Provincial CDC’s HFMD Network Laboratory for testing. At the Laboratory, RNA was extraction was carried out using a commercial viral nucleic acid extraction kit (Geneaid Biotech Ltd., New Taipei City, Taiwan) according to the manufacturer’s instructions. Real-time PCR was performed using a commercially available kit (Bioperfectus, Jiangsu, China) according to the manufacturer’s instructions. The specifications for specimen collection and real-time RT-PCR are outlined in and were performed in accordance to the requirements detailed in the chapter on HFMD specimen collection and testing technology in the “Foot and Mouth Disease Control and Prevention Guide” (2009).

The data from the March 2010 to October 2012 sample collection were used to establish the test equation, and the data from the November 2012 to October 2013 collection were used for the validation equation.

### Statistical analysis

The SPSS17.0 statistical software package was used to conduct all statistical analyses. Comparisons of the rates for viral positivity were performed by the chi-squared (χ2) test and the trends in the relationship between the variables were analyzed by linear regression modeling. Predictions were determined by combining the results with professional knowledge. The threshold for statistical significance was set as an α value of less than 0.05; for multiple pairwise comparisons, the α value was set as 0.05 per number of comparisons.

The data for the EV71 detection rate in mild cases that were detected between November 2012 and October 2013 were substituted into the equation in order to obtain the forecast value for the severe case proportion. To assess the validity of the proposed equation, the goodness-of-fit was determined and the mean absolute percentage error (MAPE) between the forecast value and the actual value was calculated using the following equation:
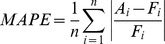



where A_i_ was the actual value and F_i_ was the forecast value.

## Results

### Rate of etiologic agent detection among all HFMD cases

Between March 2010 and October 2012, 23,297 clinically diagnosed HFMD cases were collected for analysis of pathogen presence. Viral positivity was found for 14,725 (63.21%) of the cases, including 7,419 (50.14%) with positivity for EV71, 2,629 (20.06%) with positivity for CoxA16, and 4,409 (29.80%) with positivity for other intestinal viruses. The detailed data of viral detection and distribution are presented in Table 1.

**Table 1:** Etiologic distribution of viruses detected in HFMD cases during March 2010 to October 2012 in Hunan Province.

Year	Case number	Positive cases	Positive rate, %	EV71 positivity		CoxA16 positivity		Other intestinal viruses positivity	
				Case number	Positive constituent ratio, %	Case number	Positive constituent ratio, %	Case number	Positive constituent ratio, %
2010	6937	4475	64.51	2431	54.30	713	15.93	1333	29.77
2011	7664	4410	57.54	1518	34.38	1514	34.28	1384	31.34
2012	8696	5840	67.16	3470	58.77	742	12.57	1692	28.66
Total	23297	14725	63.21	7419	50.14	2969	20.06	4409	29.80

### Statistical correlation of EV71 detection and case severity

The viral detection rate of EV71 was subjected to stratification analysis according to case severity (mild, severe, and fatal) in order to determine if identification of EV71 is dependent up on case severity (Table 2). The positive detection rate of EV71 was found to increase in conjunction with an increase in disease severity of HFMD cases (25.78% in mild cases, 52.20% in severe cases, and 84.18% in fatal cases). Furthermore, comparison of all three types of case severities (mild, severe and fatal) were tested for dependence on the EV71 detection rate and the relationship was found to be statistically significant (χ^2^ = 397.564, p<0.05). Multiple comparisons between individual case outcomes also showed statistical differences (test standard value = original detection value/comparison times). For pairwise comparisons among multiple groups, the calculation used was α’ = α/N, N = n(n-1)/2, wherein was the tested group number, (namely, α’ = 0.05/3 = 0.017). The statistical comparison between EV71 mild cases and severe cases yielded a χ^2^ of 340.994 and a p of 0.000, while the comparison between severe cases and fatal cases yielded χ^2^ = 25.115 and p = 0.000, and the comparison between mild cases and fatal cases yielded χ^2^ = 66.278 and p = 0.000.

**Table 2:** Etiologic distribution of viral agents in HFMD cases during March 2010 to October 2012 in Hunan Province, stratified by case severity.

Type	Detection case number	Positive cases	EV71 positivity	CoxA16 positivity	Other intestinal viruses positivity	EV71 detection rate, %
Mild	18137	10870	4675	2764	3490	25.78
Severe	5002	3710	2611	203	908	52.20
Fatal	158	145	133	2	11	84.18
Total	23297	14725	7419	2969	4409	31.85

### Analysis of a direct correlation between the EV71 detection rate in mild cases and the proportion of severe case among all HFMD patients

**Figure 1:**
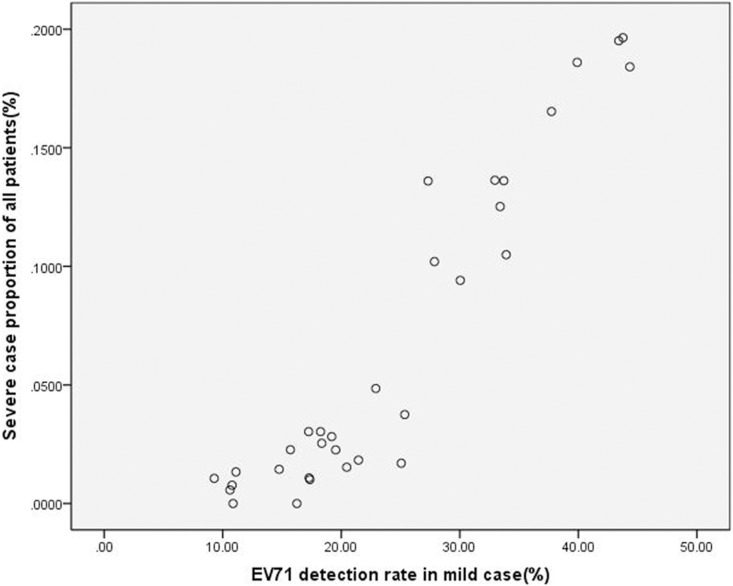
A scatter plot describing the relationship between the detection rate of EV71 in mild cases and the proportion of severe cases among all HFMD patients.

**Figure 2:**
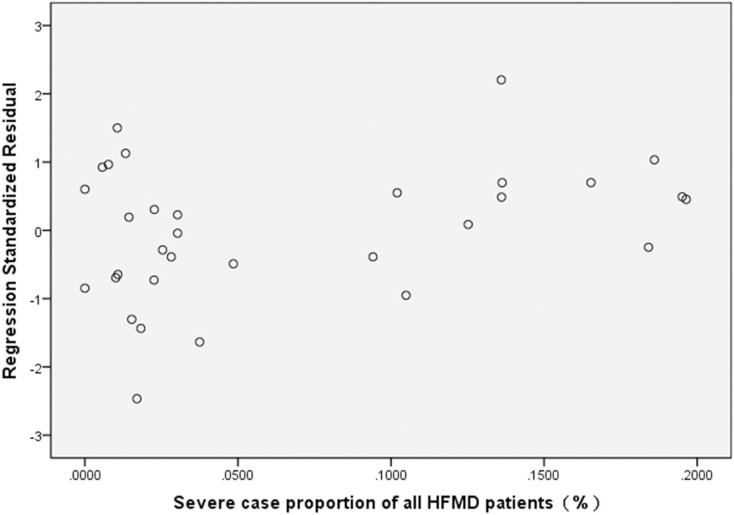
Residual plot describing the goodness-of-fit determination for the severe case proportion to the linear regression model.

The HFMD data collected from March 2010 to October 2012 that was derived from highly sensitive real time RT-PCR was utilized for linear regression modeling to assess the existence of a statistically relevant trend between the EV71 detection rate in mild cases and the severe case proportion among all HFMD patients in each month of the study period (Table 3). For this analysis, the time unit was one month, the EV71 detection rate in mild cases (%) was set as the independent variable, the proportion of severe case among all HFMD patients (%) was set as the dependent variable, and a scatter plot was generated (Fig. 1). Meanwhile, linear regression modeling was used to calculate the regression equation. The SPSS statistical analysis indicated that a positive increasing correlation exists between the EV71 detection rate in mild cases and the severe case proportion among all HFMD patients in each month of the study period (r = 0.945, R^2^ = 0.893). The regression equation between the EV71 detection rate in mild cases (x) and the severe case proportion among all HFMD patients (y) was calculated as y = 0.006×−0.08(F = 249.218, p = 0.000). The regression model showed statistical significance, with the equation slope and constant being 0.006 and −0.08, respectively; the standard regression coefficient was 0.945 (Table 4). Residual analysis was used to determine the fit of the proportion of severe cases (dependent variable) to the regression model. The results demonstrated a relatively uniform distribution of points within the horizontal areas that fell generally between −2 and 2, indicating that the model used was appropriate (Fig. 2).

**Table 3:** Monthly EV71 detection rate of mild cases and severe case proportion among all patients for the months of March 2010 through October 2012.

2010	EV71 detectionrate of mildcase, %	Severe caseproportion, %	2011	EV71 detectionrate of mildcase, %	Severe caseproportion, %	2012	EV71 detectionrate of mildcase, %	Severe caseproportion, %
Mar	30.04	0.0941	Jan	10.87	0	Jan	15.71	0.0227
Apr	33.71	0.1361	Feb	11.11	0.0133	Feb	10.61	0.0057
May	43.76	0.1964	Mar	16.25	0	Mar	25.35	0.0375
Jun	37.73	0.1653	Apr	18.35	0.0254	Apr	44.34	0.1841
Jul	32.96	0.1363	May	21.45	0.0183	Ma	39.90	0.1860
Aug	22.91	0.0485	Jun	19.53	0.0226	Jun	43.41	0.1951
Sep	10.79	0.0077	Jul	14.76	0.0144	Jul	27.32	0.1360
Oct	9.28	0.0106	Aug	17.35	0.0101	Aug	27.86	0.1020
Nov	20.46	0.0153	Sep	17.28	0.0108	Sep	33.91	0.1049
Dec	25.06	0.0170	Oct	17.25	0.0303	Oct	33.40	0.1252
			Nov	18.25	0.0303			
			Dec	19.19	0.0282			

**Table 4:** Simple regression analysis for the correlation of EV71 detection rate of mild cases to severe case proportion among all patients on a monthly basis.

	Non-standard coefficient		Standard coefficient		
	Coefficient β	Coefficient standard error	Coefficient β	*t* value	*p* value
Constant	–0.080	0.010		–7.895	0.000
EV71 detection rate	0.006	0.000	0.945	15.787	0.000

**Figure 3:**
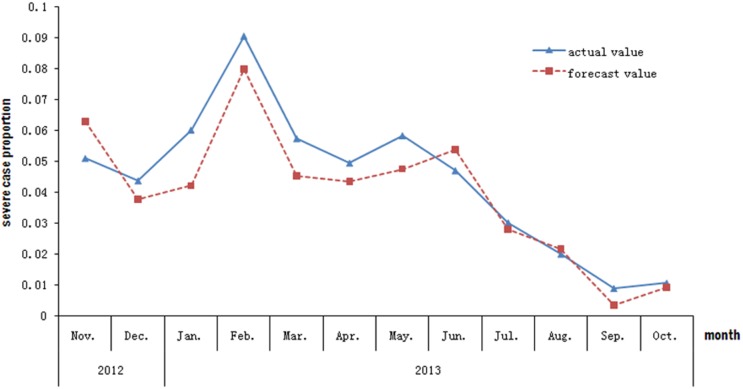
The fit-figure of the forecast value and the actual value for the data of severe case proportion from November 2012 to October 2013.

The forecast value was calculated by applying the data of the EV71 detection rate from November 2012 to October 2013 to the proposed equation. The resultant forecast value was similar to the actual value of severe case proportion, with a MAPE of 19.22%. The goodness-of-fit analysis showed that the equation provided a good fit to the real data (Fig. 3).

## Discussion

Incidence rates for HFMD severe cases remained at a high level in China for the years of 2010 through 2012 (1.58%, 1.16% and 1.04%, respectively). [12]–[14] EV71, one of the etiological agents of HFMD, is known to be especially virulent and to cause more severe disease. [12] In the current study, we also found that the detection rate of EV71 positivity was remarkably higher than that of other intestinal viruses among the severe cases. The major goal of this study was to determine if there was a correlation between the EV71 detection rate in mild cases and the proportion of severe cases among all of the HFMD patients in the study cohort. In the case that this correlation exists, we can use the data related to the detection rate – which can be obtained easily through routine surveillance – to predict HFMD severity. Our study corroborated data from previous studies that had demonstrated a pattern of increased HFMD severity following increases in the ability to detect EV71 (from less than 30% in mild cases to more than 50% in severe cases [6]; indeed, the EV71 detection rate in fatal cases in the current study reached as high as 84.18%. EV71 positive cases accounted for 71.18% of confirmed HFMD severe and fatal cases that were collected between 2010 and 2012. The dependent relationship between detecting EV71 and disease severity was also corroborated by our findings of statistically significant χ^2^ results. Overall, the data suggest that the EV71 virus is the predominant cause of severe and fatal cases of HFMD.

In the current study, we identified and validated a positive linear correlation that exists between the EV71 detection rate in mild cases and the proportion of severe cases among all HFMD patients on a monthly basis. These results demonstrate the importance of early detection of the HFMD etiologic agent to prevent disease progression. Many factors are known to affect the incidence of severe cases of HFMD. [13]–[15] Currently, the corresponding prevention and control recommendations for this disease include improving the ability to diagnose potentially severe cases before they progress as well as initiation of timely treatments (most of which are passive control measures) during mild disease presentation. [16] In contrast, there have been no systematic studies or reports published in the publicly available literature that have addressed outbreaks and which have used a significantly higher number of severe cases; in particular, none have addressed how to develop methods for predicting disease progression from mild status to the severe and/or fatal status. Establishing the correlation, as done in the current study, has served to emphasize the importance of early identification of the etiologic agent for HFMD cases in order for health facilities to intervene at earlier stages of the disease and to help prevent fatal outcomes. In addition, early detection will afford countries and/or regions the ability to more accurately forecast outbreak patterns and implement necessary prevention measures in a timely manner.
